# Valproic Acid Promotes Early Neural Differentiation in Adult Mesenchymal Stem Cells Through Protein Signalling Pathways

**DOI:** 10.3390/cells9030619

**Published:** 2020-03-04

**Authors:** Jerran Santos, Thibaut Hubert, Bruce K Milthorpe

**Affiliations:** 1 Advanced Tissue Regeneration & Drug Delivery Group, School of Life Sciences, University of Technology Sydney, P.O. Box 123, Broadway, NSW 2007, Australia; tibo.hubert@gmail.com (T.H.); Bruce.Milthorpe@uts.edu.au (B.K.M.); 2 Ecole Nationale Supérieure D’agronomie et des Industries Alimentaires (ENSAIA), Université de Lorraine, 2 Avenue de la Forêt de Haye, 54505 Vandœuvre-lès-Nancy, France

**Keywords:** adipose derived stem cells, valproic acid, protein interactions, MAPK pathway, JAK/STAT pathway

## Abstract

Regenerative medicine is a rapidly expanding area in research and clinical applications. Therapies involving the use of small molecule chemicals aim to simplify the creation of specific drugs for clinical applications. Adult mesenchymal stem cells have recently shown the capacity to differentiate into several cell types applicable for regenerative medicine (specifically neural cells, using chemicals). Valproic acid was an ideal candidate due to its clinical stability. It has been implicated in the induction of neural differentiation; however, the mechanism and the downstream events were not known. In this study, we showed that using valproic acid on adult mesenchymal stem cells induced neural differentiation within 24 h by upregulating the expression of suppressor of cytokine signaling 5 (SOCS5) and Fibroblast growth factor 21 (FGF21), without increasing the potential death rate of the cells. Through this, the Janus Kinase/Signal Transducer and Activator of Transcription (JAK/STAT) pathway is downregulated, and the mitogen-activated protein kinase (MAPK) cascade is activated. The bioinformatics analyses revealed the expression of several neuro-specific proteins as well as a range of functional and structural proteins involved in the formation and development of the neural cells.

## 1. Introduction

Regenerative and translational medicine is a rapidly expanding area made possible by the availability of an abundant source of stem cells, particularly autologous adult mesenchymal stem cells acquired from lipoaspirates termed adipose derived stem cells (ADSCs). The application of autologous ADSCs to neural regeneration and repair therapies is of great interest due to the potential to reverse or limit the exacerbation of injuries that have severe effects on the quality of life while avoiding rejection [1].

Several studies have explored the effect of small molecule chemical inducers on the potential to drive neurogenic differentiation in stem cells. At optimized concentrations and short treatment times, chemicals such as beta-mercaptoethanol (BME) and dimethylsulfoxide (DMSO), have shown the potential to induce a structural and molecular phenotype in stem cells that resemble differentiating neural cells [2,3]. Not surprisingly, these molecules had negative effects inducing a range of stress and apoptotic markers with an increasing treatment time frame. Alternatively, less harsh chemicals with similar actions, such as butylated hydroxyanisole (BHA), retinoic acid (RA) and other chemical derivatives, have also shown a marked capacity for improving neural differentiation while decreasing cellular stress and death [4,5,6,7]. Valproic acid (VPA), as a focus molecule, has garnered some attention in neurogenic differentiation research, and was previously explored in certain stem cell types; however, the extent of the differentiation was not completely elucidated [8,9].

Valproic acid (VPA) is a short-chain fatty acid that is well known as a histone deacetylase (HDAC) inhibitor [10]. It is an established drug in epilepsy therapy and can be used clinically as an anticonvulsant and a mood stabilizer [11]. Its proprieties on adult neuron cells are known and its actions on transcription have been previously studied at variable concentrations in vitro [12]. Studies have proven that VPA can affect the proliferation and the differentiation of neural crest progenitors and hippocampal neural stem cells [13]. Furthermore, VPA was shown to increase white matter repair and neurogenesis after a stroke by supporting the survival and new growth of oligodendrocytes, as well as myelination and axonal density [14]. The effect of VPA inducing differentiation in other stem cell types has been studied to a lesser extent. 

VPA is minimally cytotoxic and biologically relevant. The induction by VPA of placental mesenchymal stem cells toward neuronal differentiation has been analysed using the criteria of altered cell morphology, reduced proliferation, and the expression of marker genes [13]. VPA treatment for up to ten days induced profound changes in cell morphology, which were characterized by less tightly packed cells within the colonies and the generation of long filamentous structures [8]. VPA has a definite effect on stem cells and induced morphological changes that progressed to a stage of preneuronal-like cell. 

Further studies showed that VPA influences the proliferation and differentiation of neuronal cells by expressing a small set of specific markers [15]. However, little is known about the downstream events. The role of VPA in protein expression involved in the cell cycle and neuronal differentiation was also investigated. It was shown that treatment with VPA during the progenitor stages resulted in the strong inhibition of cell proliferation and the induction of neuronal differentiation, accompanied by increases in the expression of pro-neural transcription factors and in neuronal cell numbers [15]. Furthermore, it was previously demonstrated that VPA initiated catecholaminergic neuronal differentiation. VPA launches differentiation mechanisms in sympathoadrenal progenitor cells that result in increased generation of functional neurons [9]. However, the target of VPA on the neuronal differentiation pathways is also largely unknown. A research void exists in the molecular mechanisms that play a role in ADSCs differentiation toward neuronal phenotypes.

Previous studies on how VPA affects the neural differentiation are largely based on the impact of the VPA on the transcription through inhibition of the histone deacetylases (HDACs). This study focuses on the VPA effects on ADSCs as analysed by a proteomics approach, with the aim to compare the effects of the neural induction by VPA to controls and the effects of neurobasal media B27. Media B27 supports the neural differentiation of stem cells [16,17]. Using microscopy analysis, the morphological changes of the ADSCs after induction with VPA may be tracked photographically and at specific chosen time points. The proteomic analysis provides a broader data pool of the global effect of the VPA, not only on the transcription but also directly on the different neural differentiation pathways by inducing critical interactions. Furthermore, investigation of the proteins critical in VPA induction of the cascade pathways such as MAPK/ERK and JAK/STAT may be undertaken. In addition to proteomics, a BioPlex analysis allows for the investigation of the roles of chemokines and cytokines as a complementary analytical technique. This provides more specific information on the secreted cytokines and their role in the induction of differentiation pathways in the cells [18].

## 2. Methods

### 2.1. Cell Culture

The procedures of adult human ADSCs isolation and expansion were used from Santos et al. [3] utilising cells that were cryo-stored from UTS-HREC Santos-2013000437. All donor participants volunteered through informed consent for waste lipoaspirate donation as per ethics guidelines and were de-identified for research purposes (Ethical Code: UTS-HREC Santos-2013000437, Committee: University of Technology Sydney (UTS) Human Research Ethics Committee, Date: 02/07/2013). Generally, ADSCs were cultured in T175 (Nunc, ThermoScientific, Carlsbad, CA, USA) in DMEM Glutmax/F12 Gibco, Life Technologies, Carlsbad, CA, USA) with 10% foetal bovine serum (FBS, Gibco, Life Technologies, Carlsbad, CA, USA) incubated at 37 °C at 5% CO_2_ ADSCs were passaged three to five times post isolation by stripping the cells with TrypLE Express (12604 Gibco) before being used in differentiation experiments. The cells were seeded on to 6-well plates (Nunc, ThermoScientific, Carlsbad, CA, USA) at approximately 20,000 cells/mL in 5 mL of DMEM Glutmax/F12 with FBS and maintained till 80% prior to commencing chemical induction for differentiation.

### 2.2. Chemical Induction for Differentiation

Sub-confluent ADSCs were washed twice in pre-warmed sterile DMEM Glutmax/F12 (Invitrogen). The cells were then cultured for a further 24 h in a serum-free pre-induction medium consisting of DMEM/F12 (Invitrogen), and 10% of the final concentration of the added VPA. The media was then replaced after 24 h with the neuronal inducing media consisting of DMEM/F12 (Invitrogen), and the final optimised concentrations of 0.2 mM VPA. The control cells were maintained in DMEM Glutmax/F12 Gibco, Life Technologies, Carlsbad, CA, USA) with 10% foetal bovine serum (FBS, Gibco, Life Technologies, Carlsbad, CA, USA) and a further control of ADSCs in B27 for 24 h was also maintained for further comparative analysis.

### 2.3. Cell Harvesting Sample Preparation

The cells were harvested for proteomic analysis by liquid chromatography-tandem mass spectrometry (LC-MS/MS), at the selected time points of 0, 3, 6, and 24 h post-treatment, were completed in biological and technical triplicates. Culture media was collected from each well in 2 mL Eppendorf tubes and stored at −80 °C for later Bioplex, alkaline phosphatase, and Reazurin assays. Cells were rinsed twice in 5 mL of 1× phosphate buffered saline (PBS, Merck KGaA, Darmstadt, Germany) for 5 min each at 37 °C and aspirated. Cells were then scraped into 1 mL of 1× PBS using a cell scraper (Sarstedt, Numbrecht, Germany) liberated cells were collected into an Eppendorf tube and centrifuged at 4000× *g* for 10 min. The supernatant was then discarded, and the cell pellets were stored at −80 °C till processing.

### 2.4. Alkaline Phosphatase Activity Assay

Alkaline phosphatase (ALP) is widely used as a measure of stem cell proliferative capacity as well as a marker to show pluripotency [19] and a substantial expression increase from basal states is a measure of osteoblastic differentiation [20]. From the collected conditioned media at the chosen time points, 50 μL of media was combined with 50 μL of 4-nitrophenol phosphate (*p*-NNP), the substrate for the colorimetric assay, and the absorbance was measured at 405 nm and recorded on a Tecan spectrophotometer. As ALP is continuously expressed in dividing stem cells, a relative abundance of secreted ALP can be utilized to determine the cell population proliferation in the presence of cell culture additives. Student’s t-test was used for statistical analysis, and *p*-values less than 0.05 were considered to be significant.

### 2.5. Cytotoxicity Assay 

Similarly, the cytotoxicity assay was completed in triplicate using 100 μL aliquots of the collected conditioned media from the chosen time points combined with 10 μL of Reazurin from the Alamar blue kit and incubated for 2 h at 37 °C in a clear flat bottom 96-well plate. The plate was then scanned on a Tecan spectrophotometer at a measurement wavelength of 575 nm with a 9 nm bandwidth and a reference wavelength scan at 600 nm with a 9 nm bandwidth. Absorbance vs. time graphs were generated to examine the relative cytotoxicity for each time point. Student’s t-test was used for statistical analysis, p-values less than 0.05 are considered to be significant.

### 2.6. Cytokine and Chemokine Bioplex Analysis

Bioplex analysis was performed as per Santos et al. [3] with 500 μL aliquots collected at timepoints and controls as follows: DMEM control, starve, B27 control, 0, 3, 6, and 24 h. The assay was performed with Bioplex human 27-plex (M50-0KCAF0Y Bio-Rad Laboratories, Hercules, CA, USA). The data analysis was completed in DanteR software (DanteR version 1.0.0.10. R version 2.12.0 The R Foundation for Statistical Computing, Auckland, New Zealand) [21].

### 2.7. Cell Lysate Protein Extraction Sample Preparation

The cell pellets were resuspended in 100 µL 8 M urea (Merck KGaA, Darmstadt, Germany) and 100 mM ammonium bicarbonate (Merck KGaA, Darmstadt, Germany), sonicated for 10 min at 50% power at three 10 s intervals. The samples were then heated to 95 °C on a heat block for 10 min, then centrifuged for 1 min at 5000× *g*. The solution was then reduced and alkylated by adding a final concentration of 10 mM tributyl-phosphate (TBP, Merck KGaA, Darmstadt, Germany) and 20 mM acrylamide (Merck KGaA, Darmstadt, Germany), then vortexed and spun down on a mini-centrifuge (Qik Spin QS7000 Edwards Instruments) at 2000× *g* for 2 s. The samples were incubated for 90 min at room temperature then quenched with a final concentration of 50 mM dithiothreitol (DTT, Merck KGaA, Darmstadt, Germany)) and again vortexed and spun down on a mini-centrifuge at 2000× *g* for 2 s. The samples were then diluted 1:8 in 100 mM ammonium bicarbonate. We then added 0.5 µg of trypsin to digest at 37 °C for a minimum of 12 h. The samples were then desalted using SiliaprepX SCX SPE solid phase extraction columns (Silicycle, Quebec City, Canada). The peptide concentration was determined using the Pierce quantitative colorimetric peptide assay (Thermofisher Scientific, NSW, Australia) and prepared for LC-MS/MS analysis.

### 2.8. Liquid Chromatography-Tandem Mass Spectrometry 

An Acquity M-class nanoLC system (Waters, USA) was used, loading 5 µL of the sample (1 mg) at a rate of 15 mL/min for 3 min onto a nanoEase Symmetry C18 trapping column (180 mm × 20 mm). It was then washed onto a PicoFrit column (75 mm ID × 250 mm; New Objective, Woburn, MA, USA) packed with Magic C18AQ resin (Michrom Bioresources, Auburn, CA, USA). The column was then eluted of peptides into the Q Exactive Plus mass spectrometer (Thermofisher Scientific, NSW, Australia) using the following program: 5%–30% MS buffer B (98% Acetonitrile +0.2% Formic Acid) over 90 min, 30%–80% MS buffer B over 3 min, 80% MS buffer B for 2 min, 80%–5% for 3 min. The peptides that were eluted were ionised at 2000 V. A data dependant MS/MS (dd-MS2) experiment was performed, with a 350–1500 Da survey scan was performed at a resolution of 70,000 m/z for peptides of charge state 2+ or higher with an Automatic Gain Control (AGC) target of 3 × 10^6^ and a 50 ms maximum injection time. The top 12 peptides were selectively fragmented in the Higher-energy collisional dissociation (HCD) cell using a 1.4 m/z isolation window, an AGC target of 1 × 105 and a 100 ms maximum injection time. The fragments were scanned in the Orbitrap analyser at a resolution of 17,500 and the product ion fragment masses were measured over a 120–2000 Da mass range. The mass of the precursor peptide was then excluded for 30 s. 

### 2.9. Mass Spectrometry, Protein Identification and Data Analysis

The MS/MS data files were searched against the Human Proteome Database and against common contaminants using Peaks Studio version 8.5 with the following parameter settings: fixed modifications: none; variable modifications: propionamide, oxidised methionine, deamidated asparagine; enzyme: semi-trypsin; number of allowed missed cleavages: three; peptide mass tolerance: 30 ppm; MS/MS mass tolerance: 0.1 Da; charge states: 2+, 3+, and 4+. The search results were filtered to include peptides with a −log10P score (related to P-value) determined by the false discovery rate (FDR) of less than 1%, where the score indicates that the decoy database search matches were less than 1% of the total matches. Each condition was made up of the biological replicates that were treated at the same time, run in triplicate. Data analysis was completed in Microsoft Excel 365, Peaks version 8.5, DanteR (DanteR version 1.0.0.10. R version 2.12.0 The R Foundation for Statistical Computing, Auckland, New Zealand) [21], Cytoscape (version 3.7.1, Cytoscape Consortium, Seattle, WA, USA) [22].

## 3. Results

### 3.1. Live Cell Temporal Microscopy during Neurogenic Induction Differentiation of Human ADSCs

**Figure 1:**
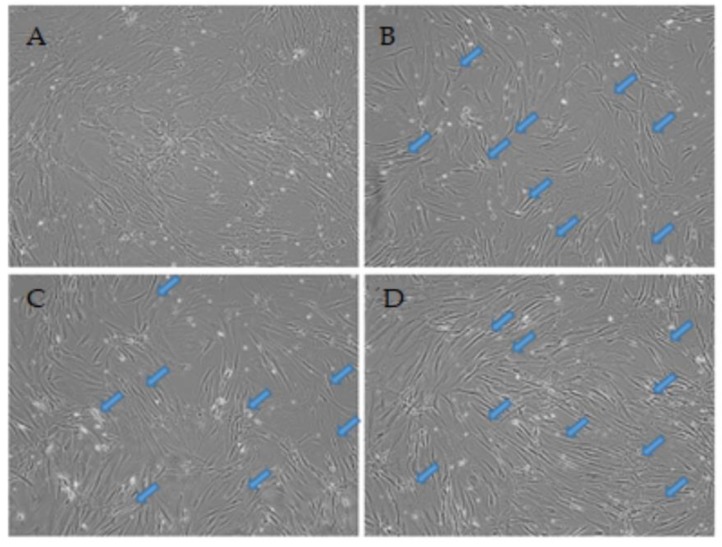

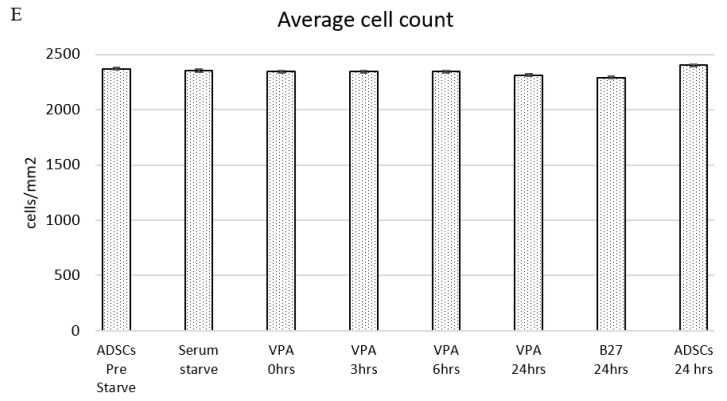
Live cell images of the temporal differentiation of human adipose derived stem cells (ADSCs) induced with 0.2 mM valproic acid (VPA) at (**A**) 0 h, (**B**) 3 h, (**C**) 6 h, and (**D**) 24 h at 10× magnification. The cellular morphology changes rapidly through the time points, with cells adopting a more slender and bipolar orientation with neurite extensions (arrows). (**E**) Average cell count across the treatments including all controls with standard error bars. Relatively minimal changes in numbers are shown across all treatments. A Student’s t-test revealed no significant change in cell numbers.

Live cell microscopy is a vital procedure to track cellular morphologies over time during differentiation. The physical attributes in the cell shape and formation of substructures on cells can specify the health status and stage of differentiation relative to the treatment [23]. The ADSC control (Figure 1A) shows non-induced cells at passage 3 at 0 h with a typical morphology and diffuse growth with wide cell bodies. Figure 1B–D shows the same field of view through time points 3, 6, and 24 h, respectively, displaying the temporal changes occurring in the identical field of view. The treatment with VPA induced morphological and phenotypical changes in the ADSCs resembling differentiating or pre-neural cells. Generally, over time, the cells structural rearrangement shows an adopted bipolar stretched out shape displaying a condensed cell membrane around the nuclear region within the first 3 h. Furthermore, the appearance of dendrite-like structures is also increasingly more apparent from the 6 h time point and are marked with arrows.

By 24 h, the majority of the cells now display signs of morphological shifts from the control, with a large majority of cells displaying uniform structural changes producing long-extensions between cells and neurite-like outgrowth on some cells. The cell population has remained relatively unchanged across all time points compared to the control, as shown in the average cell counts in Figure 1E. This indicates that minimal to no damage or death due to stress and apoptosis is present in the treated cells.

**Figure 2:**
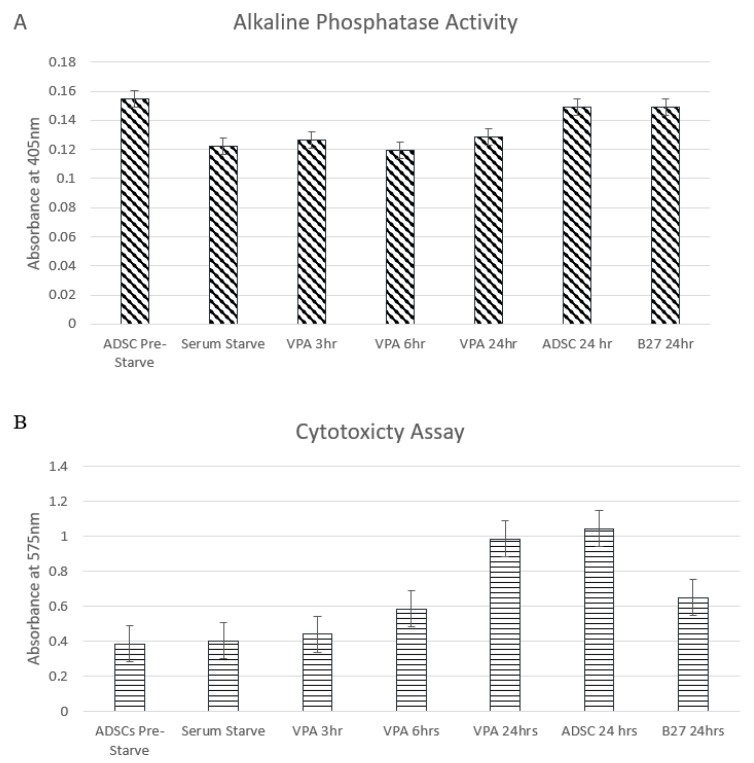
(**A**) shows the alkaline phosphatase (ALP) activity of cells in each treatment over time. ALP activity decreases marginally after serum starve and remains at relative levels through the treatment time points with standard error bars. There was no significant change seen in the t-test of data. (**B**) shows the cytotoxicity assay over time as cellular stress was detected by the Reazurin level with standard error bars. Early treatment time points are equivalent to the pre-starved ADSCs prior to treatment. The levels increase from 6 h and 24 h; however, they remain below ADSCs control at 24 h and B27 control at 24 h post media change. There is no statistical significance determined by the t-test.

Supporting the observation of minimal to no damage or death are the graphs in Figure 2 displaying alkaline phosphatase activity on y-axis Figure 2A and Reazurin cytotoxicity assay on y-Figure 2B. The ALP column graphs show that the ALP activity decreases marginally in the serum starved cells and for the post VPA treated cells, the similar expression range of ALP confirms the cells are not experiencing osteogenic differentiation secretion levels and that the ALP secretion is maintained within basal levels. The Reazurin cytotoxicity assay line graph shows that the levels at 3 h post treatment are similar to the pre-treatment values. The stress is marginally increased at 6 and 24 h however this is lower than the ADSC media change at 24 h, confirming that the VPA treatment has a minimal stress and cytotoxic effect on ADSCs at the treated concentrations relative to controls. Student’s t-test analysis revealed no significant change in cell numbers.

### 3.2. Cytokine and Chemokine Bioplex Analysis

**Figure 3:**
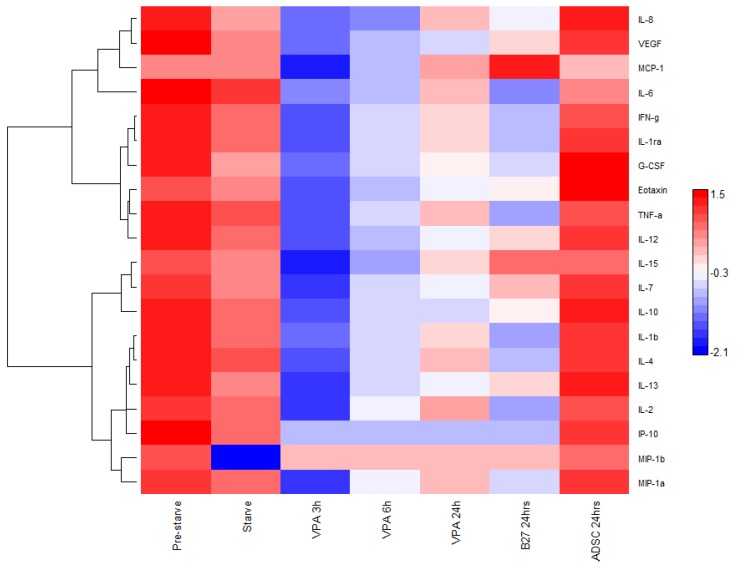
A Bioplex heat map of the log10 measure of cytokines and interleukins expressed in ADSCs in control DMEM media pre FBS starved; starved and in DMEM after 24 h or treated with B27 or VPA over time. Hierarchical clustering software and a Euclidean test. Red: expression above median; Blue: expression below the median; White: median expression across sample.

Cytokines and chemokines are multifunctional molecules with a plethora of roles based on their cellular location. Briefly, some of their roles include, pro-inflammation, anti-inflammation, intracellular signalling, intercellular signalling, response to external stimuli, induction or response to protein cascades, and response or guidance of differentiation in cells. Their importance in stem cell differentiation and their response to external stimulation is paramount to clarifying their role in response to VPA. To investigate the relative quantitative changes in the expression and secretion of cytokines and chemokines, the Bioplex multiplex immunoassay was used to simultaneously quantify the molecules in each sample collected from controls and VPA induced time points at 0, 3, 6, and 24 h (Figure 3). The molecules measured were Eotaxin, Granulocyte-colony stimulating factor (G-CSF), Interferon gamma (IFN-γ), Interleukin IL-1β, IL-1ra, IL-2, IL-4, IL-6, IL-7, IL-8, IL-10, IL-12 (p70), IL-13, IL-15, Interferon gamma-induced protein 10 (IP-10), Monocyte Chemoattractant Protein-1 (MCP-1), Macrophage Inflammatory Proteins1 alpha (MIP-1α), MIP-1β, Tumor necrosis factor alpha (TNF-α) and Vascular endothelial growth factor (VEGF). From the 3 h time point post VPA treatment of the ADSCs, there were lower concentrations of all measured molecules with no expression levels of IP-10 and MIP-1b detected through any successive time points. While most of the other molecules regain some cumulative presence post VPA treatment; IL-1β, IL-6, and TNF-α demonstrate a marked decrease in levels below the non-treated samples and remain low through all time points with relatively closer levels to the B27 treated cells. 

### 3.3. Proteome Comparisons of VPA Treated Cells

Each cell treatment was conducted in biological duplicates of tissue cultures. Subsequently, each sample was analysed in technical triplicates by mass spectrometry. This allowed for up to six analysis points for each treatment. This was completed for an increased stringency identification and analysis. Mass spectrometry data compilation (Table 1) identified, at the 95% confidence cutoff, 2067 unique proteins matched from 20,011 distinct peptides derived from a 344,510 total spectra count (Table 1). There was an average of 2.51 peptides matched per protein with an average of 10% sequence coverage. Proteins were removed from the analysis if they were identified by less than two quantifiable peptides per protein. The proteins analyzed were uniquely detected per time point or expressed successively through two or more treatment time points. 

**Figure 4:**
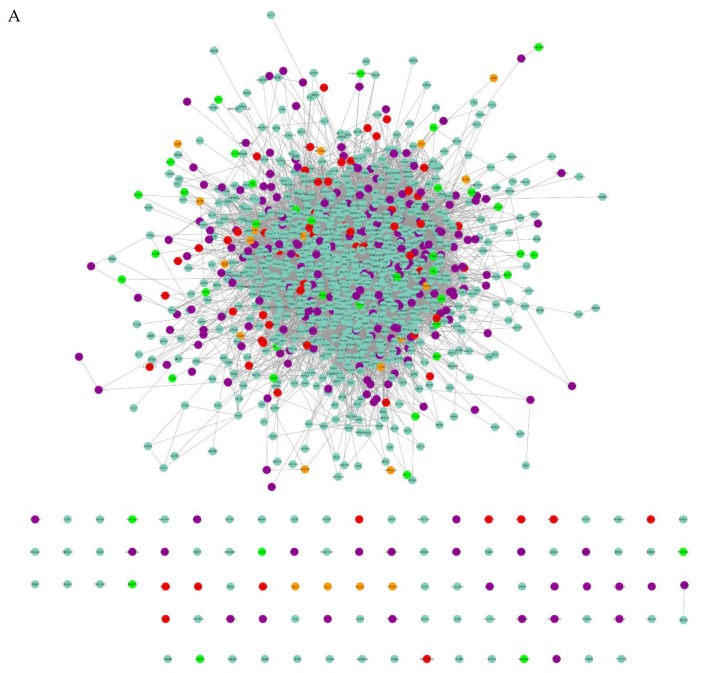

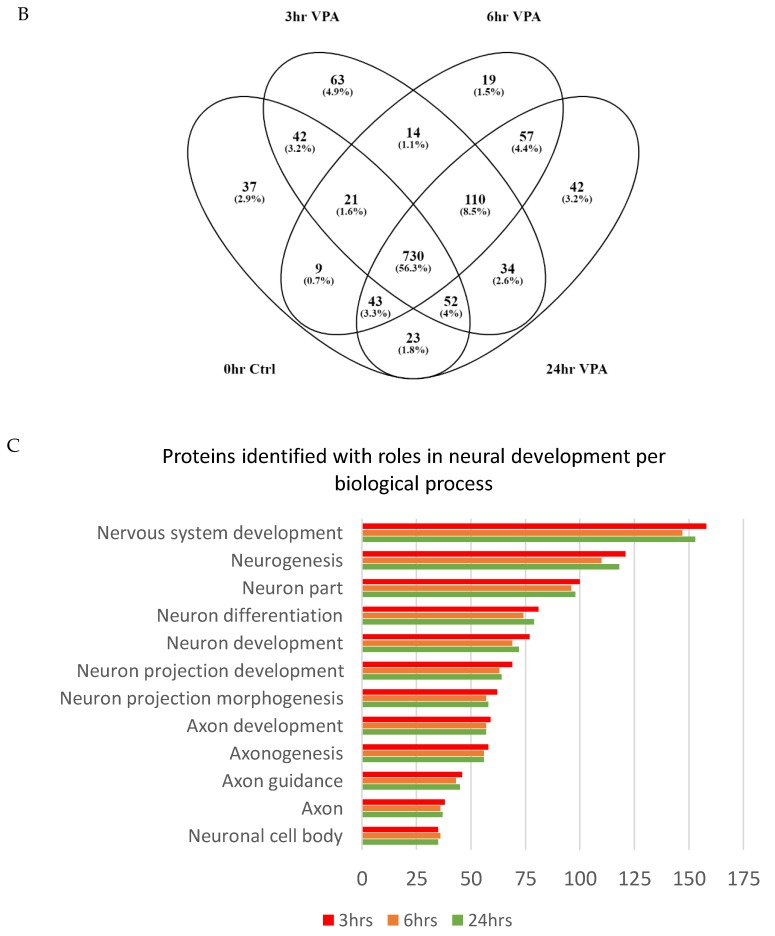
(**A**) Cytoscape protein interaction network graph of proteins identified by mass spectrometry. Blue—ADSC unique, Violet—occurs in two or more time points, Red—3 h unique expression, Orange—6 h unique expression, and Green—24 h unique expression. (**B**) Venn diagram displays breakdown of protein numbers unique and shared between time points. (**C**) Gene ontology biological process analysis of the proteins expressed in Red—3 h, Orange—6 h, and Green—24 h shows a high percentage of proteins linked to neural, neuron, or axon development.

Interaction network analysis (Figure 4A) using the Cytoscape overlays, the 0, 3, 6 and 24 h VPA treatment time points combining duplicates and removing proteins with less than two peptides, and post colour-coding for unique and shared proteins, the network displays 1256 proteins with 12,771 interactions. This was completed to locate the date interaction hubs between time points and to visualize the protein pathways. In Figure 4A, the nodes are Blue—ADSC unique, Violet-occurs in two or more time points, Red—3 h unique expression, Orange—6 h unique expression, and Green—24 h unique expression. The Venn diagram breakdown (Figure 4B) displays the number of proteins that are unique and shared between each time point as well as percentage of total proteins analysed in the network. The proteins expressed in the ADSC VPA treated time points overlap with only the VPA treated ADSCs total to 3 h—221 proteins, 6 h—200 proteins, and 24 h—243 proteins. The gene ontology analysis of the proteins expressed in the VPA treated ADSCs is graphed in Figure 4C. There are 150 proteins involved across several biological process ontologies aligned with neuron and axon development, projection, differentiation, and cell body morphogenesis.

### 3.4. Pathway Analysis of Gene Ontology Clustered Proteins

**Figure 5:**
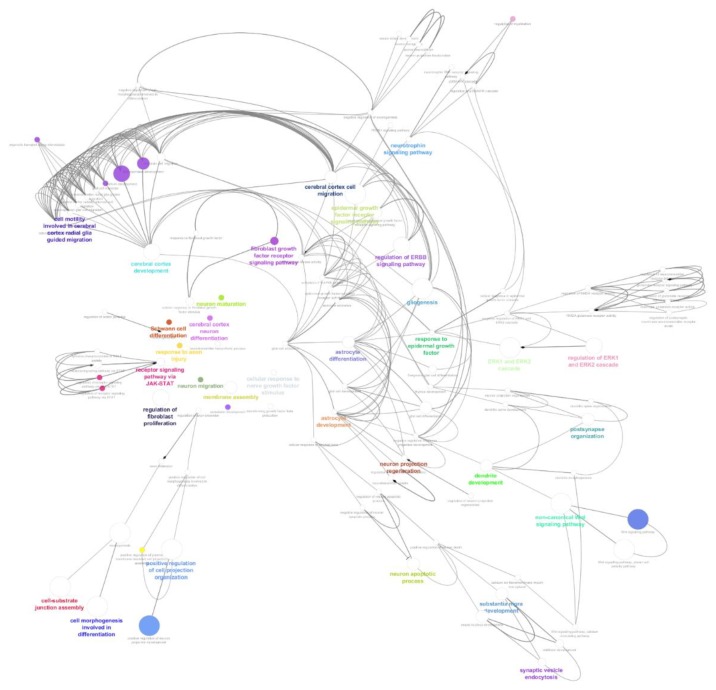
The ClueGo analysis of clustered proteins within each GO node for biological process interaction and signalling pathway analysis involved in the VPA treated cells. Node sizes are relative to number of proteins in dataset identified in that GO. Coloured nodes and labels represent important GO terms in the network.

The above-mentioned neural-related proteins identified in gene ontology biological process categories were analysed in ClueGO (version 2.5.4) for group clustering and interaction pathway process analysis (Figure 5). Graphing only the neural-related proteins allowed for a concise interaction map of the probable roles played in the differentiated process as well as links in the signalling pathways. Table 2 breaks down the gene ontology type, number of proteins identified in this dataset as well as the GO term *p*-value Bonferroni step down to validate multiple pair wise tests.

**Figure 6:**
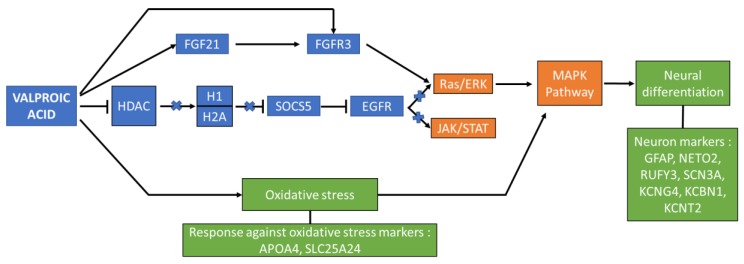
Reductive schematic of the proposed VPA induction pathway in the treatment of ADSCs toward neural differentiation.

Figure 6 is a reductive schematic derived from the previous ClueGO graph; it presents the proposed key proteins (summarized in Table 3) and possible summary pathway involved in VPA’s action in directing signalling and differentiation in the treated ADSCs. VPA is known to be an HDAC inhibitor, by this direct interaction, HDAC secondary interaction with H1 and H2A is closed. These two proteins have further downstream affects in regulating the RAS/ERK and JAK/STAT pathway via EGRF signalling. By closing this loop, the FGF signalling pathway is upregulated by direct and indirect interactions of VPA which promote the MAPK1 expression and activity. This is a gateway control for initiating the MAPK pathway, which is involved in several neural differentiation lineages. VPA also has an activity in oxidative stress which also acts as a dualistic interaction hub for MAPK pathway promotion.

## 4. Discussion

The variety of neural related applications of Valproic acid, including its use as an anticonvulsant to use in treating disorders, such as epilepsy, bipolar, Schizophrenia, Parkinson’s, and stroke, holds significant value in its role in directing in vivo neural cell modulation and repair. In vitro studies have observed a VPA effect on several factors in molecular responses of stem cells treated with the chemical, indicating shifts toward neural-like outgrowth with the expression of specific markers and morphological ques associated with differentiation. In this study we aimed to observe and measure the effect of VPA on the wide changes over chosen time points of the proteome of adult stem cells to gain a global understanding of the early mechanisms that are involved in the VPA induction of stem cells.

### 4.1. Cellular Morphology 

The microscopy analysis exhibits the first evidence of differentiation. VPA induces morphological changes on the ADSCs within 3 h, and the cells show long cytoplasmic extensions and become thicker relative to the untreated cells. This indicates that VPA may influence cytoskeletal modifications. Dendrite-like structures appear from 6 h provides further evidence that ADSCs are taking on a neuronal differentiation path. At the final time point, examined at 24 h, the treated cells have a vastly changed appearance from the original form. Much of the population appears slender and has a directed polar outward growth pattern. Furthermore, the dendrite extensions are present in most cells. VPA does not have an influence on the death rate, the cell population has had minimal variation over the treatment time points as opposed to studies with other small chemicals, such as BME and DMSO [2,3,23]. The cell counts and subsequent ALP and Reazurin assays support the stability of the cell culture population with nominal changes over time. 

### 4.2. Secreted Molecules Role in Signalling Pathways Controlling Neural Differentiation

The pleiotropic nature of secreted cytokines assists in the molecular signalling and pathway modulation across various systems in almost all cell types. They are particularly interesting due to the multifunctional roles within cellular responses and differentiation, by primarily promoting or closing pathways as well as regulating the expression of specific cascades leading to the release of neurotrophic factors in the case of neural differentiation. Tracking their expression patterns from treated cells utilizing the Bioplex system, allows for a relative quantification and group clustering to identify trends between multiple molecules.

In the Bioplex results, it is noticeable that for almost all cytokines, post VPA treatment, the quantified amounts are reduced from that time point. The levels seem to increase by the final time point with variable expression patterns that are cytokine specific. 

We found that VPA has effects on every cytokine measured within this study, thus it has a large action spectrum of modulating cytokine expression, however, is very specific for targets such as IP-10 and MIP-1b. For these two cytokines, IP-10 and MIP-1b, VPA definitively stops their expression upon treatment. IP-10 is not detected after the first control time point across all replicates. This may indicate that VPA has an irreversible action on these particular cytokines. Whereas, the other cytokine trends indicate possible reversible action as variable expression patterns are viewed across time points. IP-10 and MIP-1b have known interactions and roles with the JAK/STAT protein pathway [24,25]. The JAK/STAT canonical pathway is a system of linked interactions involved in the phosphorylation of tyrosine residues in receptors creating active binding sites for proteins with Src-Homology (SH2) domains. 

In this receptor—SH2 activation the ligand proteins are translocated to the cell nucleus to initiate the transcription of genes that are vital in development, immunity and oncogenesis [26]. MIP-1b and STAT-1 protein interaction were previously implicated in the development of human glial cells and astrocytes [27]. IP-10 has also been shown to play a critical role in the maintenance of maturing astrocytes in vitro [28]. The limitation of these two molecules expression and subsequent interactions could limit the astroglial lineage commitment and instead promote neuronal development via the MAPK pathway. Supporting this, astrogliogenesis has been noted to require a high expression of neuroinflammatory cytokines IL-1β, IL-6, and TNF-α during the development process [29]. 

These molecules were detected within the VPA treatments at levels substantially below the non-treated ADSC controls. Higher expression levels of IL-1β [30,31], IL-6 [32] and TNF-α [33] are known to be inhibitory or to limit to neurogenesis as well as promote cellular proliferation. Chen et al., found that increased IL-1β limited neurogenesis by co-stimulation of STAT in the JAK/STAT pathway [34]. Jian et al., noted that the repression of the STAT occurs by activating the MAPK pathway [35], thus supporting the dualistic nature of JAK/STAT versus MAPK. The reduced expression of the abovementioned cytokines by modulation of VPA treatment favours neurogenesis induction over astrogliogenesis.

The expression changes in cytokines involved in one or several pathways like the ERK/MAPK or JAK/STAT pathways affect, even if it is temporally, the pathways and inductive effects on the cellular cycle and the induction of differentiation.

### 4.3. Protein Expression and Interaction Pathways Affected by VPA Treatment

The proteomics and network analysis show that VPA induces the expression of a variety of neural developmental and differentiation related proteins. An interesting protein expressed due to VPA influence is SOCS5. It is found in the 3 h samples, so the VPA effect on its expression is short; however, its functional role is considerable. SOCS5 is categorised in the protein family group of suppressors of cytokine signalling. SOCS5 gene expression is repressed by histone deacetylation [36], and we know that VPA inhibits class I HDAC [11]. Thus, VPA treatment assists in increasing the expression of SOCS5 by inhibiting the HDAC. Studies proved that SOCS5 inhibits the JAK/STAT pathway [37] by decreasing STATs activation [38]. SOCS5 is also involved in the negative regulation of IL4 and IL7 signalling [38]; this is seen in this studies Bioplex results. The expression of SOCS5 could be one of the responsible elements in decreasing IL4 and IL7 expression and possibly other cytokines. Its effect may only be temporary for most of the cytokines expressions and definitive for IP-10 and MIP-1b. 

SOCS5 has also been previously annotated to interact with epidermal growth factor receptor (EGFR). EGFR primary binding partner is epidermal growth factor (EGF) which activates several signal transduction pathways including PI3K/AKT, RAS/ERK and JAK/STAT pathways [39]. The role of EGFR is particularly important for maintaining proliferative capacities in cells which is directly promotes JAK/STAT family proteins and dysregulates mTOR pathways useful in downstream neurogenesis. Furthermore, EGFR role in JAK/STAT promotes gliogenesis in direct competitive opposition to the activation of the Ras/ERK-MAPK signalling pathway [40]. The interaction of SOCS5 and EGFR is a SH-2 domain associated inhibition of EGFR, thereby the SOCS5 again has another multifunctional influence on limiting the JAK/STAT pathway [41]. SOCS5 could indirectly activate the MAPK pathway through this pathway alternation. Furthermore, the appearance of JAK3 protein in the last time points could prove that the JAK/STAT is downregulated and an intermediary protein of the cascade of reaction as JAK3 accumulates. The events conducting to this result require further investigation. By this protein interactivity the expression of SOCS5 downregulates the activation of the RAS/ERK and JAK/STAT pathways.

### 4.4. The Oxidative Stress Role in Neural Differentiation Activation 

Oxidative stress is another effect of VPA treatment, by increasing the formation of reactive oxygen species [42]. In several case studies, it was noted that increasing levels of oxidative stress can be counterproductive to cellular function; however, there is now a growing body of evidence that indicates that oxidative stress is necessary in neurogenesis [43]. A recent study by Okubo et al. [44] supports VPAs role in modulating oxidative stress by through NO-signalling. The expression of apolipoprotein A-4 (APOA4) is a marker of cellular response against oxidative stress [45]. Similarly, the calcium binding mitochondrial carrier proteins (SLC25A24) is also expressed by the cells to protect themselves from oxidative stress [46]. Within the biological process GO graph (Figure 4), these proteins were found to be database annotated [47] in cell differentiation and neuron specific ontologies, namely; generation of neurons, neurogenesis, neuron development and neuron projection development. These ontologies were found to have several subsidiary groupings, examined through the ClueGo interaction map (Figure 5), to specific neuronal developmental checkpoints supported by pathway signalling. The presence of p38 MAPKs allows for the activation of the MAPK cascade and mitotic arrest under a low level of oxidative stress, which has also been reported by Kurata et al. [48]. VPA’s role in p38 MAPK expression and the subsequent activation of the MAPK cascade is a supplementary route over the Ras/ERK signalling forbearer activation of MAPK and neurogenesis pathways. VPA creates an environment with low oxidative stress triggering the ADSCs to activating the MAPK cascade.

### 4.5. Functional Roles of Identified Neural Proteins in VPA Treated MSCs

Studies show that VPA promotes the production of Fibroblast growth factor 21 (FGF21) while suppressing HDAC [49]. FGF21 expression in this study was noted onward from the 6 h time point FGF21 is a regulator of metabolic processes and has strong links to neurogenesis and neuron maturation and myelination [50]. Its receptor FGFR3 is expressed at 3 h, 6 h, and 24 h. Studies proved that following the FGF21 activation of FGFR, the MAPK pathway is initiated, involving phosphorylation of ERK 1 and 2 [51]. In the ClueGo analysis, the FGFR signalling pathway is proximal to several downstream processes, particularly in cerebral cortex development and neuron maturation. In a recent study by Shahror et al. [52] it was shown that MSCs over expressing FGF21 transplanted to a mouse model enhanced neurogenesis and recovery. Furthermore, their results also show the FGF21 promoted maturation of the hippocampal neurons. The expression of the FGF21 and FGFR3 post VPA treatment of the stem cells is useful indicator of the neurogenic potential of VPA and MSCs.

A pertinent dataset, supportive of neuronal induction and differentiation, is the presence of neuronal markers, which appear from the first treatment time point. Table 2 presents a list of identified proteins and their correlative biological process in neurodevelopment according to gene ontology. Exploring several key markers and their interaction partners allows for a greater view of the molecular changes occurring during treatment and differentiation. The early expression of glial fibril associated protein (GFAP), neuropilin and tolloid like 2 (NETO2), and RUN and FYVE Domain Containing 3 (RUFY3) are particularly interesting. GFAP is known to be expressed in developing central nervous systems, and is particularly expressed in glia, astrocytes, neural stem cells, and neuron progenitor cells, with its expression declining in mature neurons [53]. 

It plays a vital role in neuron development, and is classed in the GO category of neuron projections along with its GO category and interaction partner, Thy1-membrane glycoprotein (THY1). The interaction of these two proteins are thought to guide cell-to-cell extension in the early phases of synapse formation. The expression of NETO2 and RUFY3 were of great interest as these proteins were annotated as specifically expressed in neurons [54,55]. NETO2 interacts with several other proteins and falls in with certain ontologies related to neurotransmitter update and glutamate receptor regulation. NETO2 is also known to assist in the development of neurite outgrowth [55]. 

RUFY3 is also involved in the growth of neurons, specifically associated with the guidance of axon growth [54], fittingly, the ontologies analysis and graphs correlate its role in neuron development, the positive regulation of cell morphogenesis involved in differentiation, and the regulation of neuron projection development. Their presence in this study of treated stem cell samples eludes to the early stages of differentiation and commitment toward neuronal lineage. Some markers appear in later time points like advillin (AVIL) and proteins involved in voltage-gated channel and that are specific in generating the neuronal action potential, such as SCN3A, KCNG4, KCBN1 and KCNT2. AVIL is involved in neuron development by cytoskeletal organization in neuron projection and morphogenesis through its interaction with intermediary filaments and is a downstream partner in the MAPK pathway. These proteins are evidentiary developing neuron markers. Their presence in the VPA treated ADSCs shows that the cells are following a neuronal differentiation pathway.

## 5. Conclusions

In this study we presented the temporal treatment of VPA on ADSCs and the proteomic, Bioplex, interaction network and pathway analysis. We found that VPA induces a cascade of reactions using its different properties. Inhibiting HDACs, it promotes the expression of SOCS5, which downregulates the JAK/STAT pathway. In this way, the glial differentiation is silenced, and the neuronal differentiation can be promoted. 

We demonstrated that VPA activates the MAPK cascade by creating a low oxidative stress and by upregulating the expression of FGF21. However, the ERK pathway is downregulated by SOCS5 as well; however, this can operate as a regulator of the activation of the ERK/MAPK cascade. Specific neuronal markers are found in the induced ADSCs and their expressions confirm that VPA induced a neuronal differentiation pathway on the ADSCs.

This study opens a new opportunity to further differentiate ADSCs into neurons using a chemical that is already used clinically. VPA can be a key to finding new neuro-regenerative methods.

## Figures and Tables

**Table 1:** The number of proteins and peptides identified after liquid chromatography-tandem mass spectrometry analysis of ADSCs and ADSCs treated with VPA.

Confidence Cut-off	Proteins Detected	Proteins Before Grouping	Distinct Peptides	Spectra Identified	%Total Spectra
>2.0 (99)	622	765	8286	313,119	66.8
>1.3 (95)	2067	2269	20,011	344,510	67.7
>0.47 (66)	2334	2460	34,492	406,322	68.5

**Table 2:** The gene ontology of proteins associated in the neural-related ClueGO interaction and signalling network map.

GO Term	GO:ID	Number of Proteins	Associated Proteins Found	Percentage of Associated Proteins in GO	Term P-Value
organelle transport along microtubule	GO:0072384	5	[CDC42, LAMP1, SUN1, SYNE2, UCHL1]	4.72	9.03 × 10^−3^
regulation of NMDA receptor activity	GO:2000310	3	[DLG1, HSPA8, MAPK1]	5.56	2.70 × 10^−2^
response to fibroblast growth factor	GO:0071774	9	[CCN2, FGF21, FGF3, FGFR3, MAPK1, POSTN, PTPN11, TFAP2C, THBS1]	4.95	3.59 × 10^−4^
membrane assembly	GO:0071709	6	[ANXA2, CAV1, FLOT1, MAPK1, PDCD6IP, SPTBN1]	11.54	3.57 × 10^−5^
cell-substrate junction assembly	GO:0007044	14	[BMX, CTTN, FN1, FYN, LAMB2, LRP1, PLEC, PPM1F, PTPRK, RAC1, RHOA, ROCK1, THBS1, THY1]	10.22	1.08 × 10^−9^
transforming growth factor beta production	GO:0071604	3	[ITGAV, MTCO2P12, THBS1]	5.88	2.32 × 10^−2^
regulation of myelination	GO:0031641	3	[DLG1, SOS1, TYMP]	6.12	2.09 × 10^−2^
positive regulation of cell projection organization	GO:0031346	24	[AP2A1, ARPC2, BMX, CDC42, DDX21, FN1, FSCN1, FYN, HSPA5, HSPA8, ITGA3, KDM1A, LAP3, LRP1, NME2, PDCD6IP, PICALM, PSEN1, RAB21, RAC1, RHOA, SCARB2, SOS1, TMEM30A]	4.60	1.90 × 10^−8^
cellular response to nerve growth factor stimulus	GO:1990090	3	[ARF6, HSPA5, PDCD6IP]	4.05	5.94 × 10^−2^
cellular response to epidermal growth factor stimulus	GO:0071364	5	[EGFR, GSTP1, PDCD6IP, PTPN11, TFAP2C]	6.33	2.60 × 10^−3^
neuron projection regeneration	GO:0031102	5	[GFAP, LAMB2, LRP1, SOS1, THY1]	6.94	1.73 × 10^−3^
action potential	GO:0001508	8	[CACNB3, CAMK2D, CAV1, DLG1, KCNB1, MYH14, SCN2A, SCN3A]	4.68	1.09 × 10^−3^
regulation of receptor signalling pathway via STAT	GO:1904892	8	[CAV1, FGFR3, FYN, HGS, IL3, JAK3, KLK3, TFRC]	4.26	1.99 × 10^−3^
neurotransmitter uptake	GO:0001504	3	[FLOT1, GFAP, PSEN1]	4.92	3.69 × 10^−2^
cellular response to fibroblast growth factor stimulus	GO:0044344	9	[CCN2, FGF21, FGF3, FGFR3, MAPK1, POSTN, PTPN11, TFAP2C, THBS1]	5.26	2.26 × 10^−4^
cellular response to amyloid-beta	GO:1904646	5	[FYN, ICAM1, LRP1, PARP1, PSEN1]	8.77	5.98 × 10^−4^
midbrain development	GO:0030901	8	[ACTB, CALM2, CDC42, HSPA5, PICALM, POTEF, RHOA, SOS1]	6.15	1.75 × 10^−4^
response to epidermal growth factor	GO:0070849	6	[EGFR, GSTP1, MAPK1, PDCD6IP, PTPN11, TFAP2C]	7.14	5.19 × 10^−4^
regulation of neuron projection regeneration	GO:0070570	3	[LRP1, SOS1, THY1]	7.14	1.39 × 10^−2^
regulation of axon extension	GO:0030516	5	[CTTN, FN1, LRP1, RAB21, RTN4]	4.46	1.13 × 10^−2^
negative regulation of ERK1 and ERK2 cascade	GO:0070373	6	[DLG1, EIF3A, GSTP1, MAPK1, PDCD6IP, TIMP3]	6.19	1.11 × 10^−3^
negative regulation of neuron apoptotic process	GO:0043524	8	[BAX, FYN, LRP1, MSH2, PSEN1, RHOA, ROCK1, TFRC]	4.04	2.74 × 10^−3^
regulation of ERK1 and ERK2 cascade	GO:0070372	18	[CCN2, DLG1, EGFR, EIF3A, FGF21, FGFR3, FN1, GNAI2, GSTP1, ICAM1, LRP1, MAPK1, MIF, PDCD6IP, PPP3CA, PTPN11, TFRC, TIMP3]	4.46	1.94 × 10^−6^
regulation of neuron apoptotic process	GO:0043523	11	[BAX, FYN, KCNB1, LRP1, MSH2, PARP1, PCSK9, PSEN1, RHOA, ROCK1, TFRC]	4.03	4.76 × 10^−4^
ERK1 and ERK2 cascade	GO:0070371	20	[CCN2, DLG1, EGFR, EIF3A, FGF21, FGFR3, FN1, GNAI2, GSTP1, ICAM1, ITGAV, LRP1, MAPK1, MIF, PDCD6IP, PPP3CA, PTPN11, TFAP2C, TFRC, TIMP3]	4.56	3.60 × 10^−7^
glial cell activation	GO:0061900	3	[EGFR, LRP1, PSEN1]	4.62	4.32 × 10^−2^
telencephalon glial cell migration	GO:0022030	4	[PDCD6IP, RTN4, SUN1, SYNE2]	9.30	1.72 × 10^−3^
telencephalon cell migration	GO:0022029	7	[EGFR, PDCD6IP, PSEN1, RHOA, RTN4, SUN1, SYNE2]	7.69	1.12 × 10^−4^
cerebral cortex development	GO:0021987	11	[ATIC, BAX, EGFR, KDM1A, LRP1, PDCD6IP, PSEN1, RHOA, RTN4, SUN1, SYNE2]	6.63	5.33 × 10^−6^
cerebral cortex neuron differentiation	GO:0021895	3	[HPRT1, NCOA3, PSEN1]	7.89	1.05 × 10^−2^
forebrain cell migration	GO:0021885	7	[EGFR, PDCD6IP, PSEN1, RHOA, RTN4, SUN1, SYNE2]	7.45	1.37 × 10^−4^
cell morphogenesis involved in differentiation	GO:0000904	37	[ARPC2, BMX, CALM2, CAMK2A, CDC42, CTTN, FAM129B, FLOT1, FN1, FYN, GRB7, HPRT1, HSP90AA1, ITGAV, KRT7, LAMB2, LRP1, MAPK1, MYH9, NCOA3, PICALM, POSTN, PPP3CA, PSEN1, PTPN11, RAB21, RAC1, RB1, RHOA, ROCK1, RTN4, SOS1, SPTBN1, THY1, TUBB3, UCHL1, VAMP3]	4.03	7.39 × 10^−11^
cell motility involved in cerebral cortex radial glia guided migration	GO:0021814	3	[PDCD6IP, SUN1, SYNE2]	18.75	8.49 × 10^−4^
cerebral cortex radial glia guided migration	GO:0021801	4	[PDCD6IP, RTN4, SUN1, SYNE2]	9.30	1.72 × 10^−3^
cerebral cortex radially oriented cell migration	GO:0021799	4	[PDCD6IP, RTN4, SUN1, SYNE2]	7.84	3.24 × 10^−3^
cerebral cortex cell migration	GO:0021795	7	[EGFR, PDCD6IP, PSEN1, RHOA, RTN4, SUN1, SYNE2]	9.46	2.95 × 10^−5^
glial cell development	GO:0021782	7	[EGFR, GFAP, GSTP1, LAMB2, LRP1, PSEN1, VTA1]	4.67	2.25 × 10^−3^
dendritic spine development	GO:0060996	6	[ARF4, ARF6, CAMK2A, CDC42, MAPK1, PSEN1]	4.58	5.02 × 10^−3^
substantia nigra development	GO:0021762	7	[ACTB, CALM2, CDC42, HSPA5, PICALM, POTEF, RHOA]	10.45	1.53 × 10^−5^
pallium development	GO:0021543	13	[ALK, ATIC, ATP2B4, BAX, EGFR, KDM1A, LRP1, PDCD6IP, PSEN1, RHOA, RTN4, SUN1, SYNE2]	5.31	8.59 × 10^−6^
telencephalon development	GO:0021537	15	[ALK, ATIC, ATP2B4, BAX, EGFR, HPRT1, KDM1A, LRP1, MAPK1, PDCD6IP, PSEN1, RHOA, RTN4, SUN1, SYNE2]	4.34	1.97 × 10^−5^
epidermal growth factor-activated receptor activity	GO:0005006	4	[EFEMP1, EGFR, PSEN1, SOCS5]	9.76	1.44 × 10^−3^
NMDA glutamate receptor activity	GO:0004972	3	[DLG1, HSPA8, MAPK1]	5.26	3.10 × 10^−2^
neuron maturation	GO:0042551	3	[KDM1A, NCOA3, RB1]	4.41	4.83 × 10^−2^
ionotropic glutamate receptor activity	GO:0004970	4	[DLG1, HSPA8, MAPK1, NETO2]	4.44	2.31 × 10^−2^
activation of MAPKK activity	GO:0000186	5	[EGFR, MAPK1, PDCD6IP, PSEN1, TFRC]	4.63	9.75 × 10^−3^
MAP kinase kinase activity	GO:0004708	5	[EGFR, MAPK1, PDCD6IP, PSEN1, TFRC]	4.03	1.69 × 10^−2^
Wnt signalling pathway, planar cell polarity pathway	GO:0060071	11	[AP2A1, AP2A2, AP2B1, CDC42, PDCD6IP, PSMA1, PSMA3, PSMB9, PSMD4, RAC1, RHOA]	8.09	7.54 × 10^−7^
Bergmann glial cell differentiation	GO:0060020	3	[GFAP, MAPK1, PTPN11]	33.33	1.36 × 10^−4^
dendrite development	GO:0016358	14	[ARF4, ARF6, CALM2, CAMK2A, CDC42, FYN, HPRT1, MAPK1, PDCD6IP, PICALM, PPP3CA, PSEN1, RAB21, RHOA]	4.52	2.39 × 10^−5^
neural retina development	GO:0003407	3	[ATP2B4, PSEN1, THY1]	4.05	5.94 × 10^−2^
neurotransmitter biosynthetic process	GO:0042136	6	[ATP2B4, CAV1, HSP90AA1, ICAM1, MTCO2P12, RAC1]	4.84	3.84 × 10^−3^
Wnt signalling pathway	GO:0016055	25	[AP2A1, AP2A2, AP2B1, CALM2, CAMK2A, CAV1, CDC42, CHD8, CTNND1, EGFR, G3BP1, ITGA3, LRP1, PDCD6IP, PICALM, PLCB3, PPP3CA, PRKAA1, PSEN1, PSMA1, PSMA3, PSMB9, PSMD4, RAC1, RHOA]	4.05	1.10 × 10^−7^
gliogenesis	GO:0042063	18	[ANXA1, EGFR, GFAP, GSTP1, KRT7, LAMB2, LRP1, MAPK1, PDCD6IP, PSEN1, PSMD4, PTPN11, RELA, RHOA, RTN4, SUN1, SYNE2, VTA1]	4.81	6.45 × 10^−7^
regulation of epidermal growth factor receptor signalling pathway	GO:0042058	9	[CDC42, EGFR, EPN1, HGS, PDCD6IP, PSEN1, RAB7A, SOCS5, SOS1]	7.63	1.26 × 10^−5^
neurotrophin signalling pathway	GO:0038179	4	[CTNND1, PDCD6IP, PTPN11, SOS1]	6.45	6.53 × 10^−3^
ERBB2 signalling pathway	GO:0038128	4	[EGFR, GRB7, HSP90AA1, SOS1]	9.52	1.58 × 10^−3^
regulation of neurotransmitter uptake	GO:0051580	3	[FLOT1, GFAP, PSEN1]	12.50	2.86 × 10^−3^
p38MAPK cascade	GO:0038066	4	[DLG1, PDCD6IP, SOS1, TFAP2C]	6.56	6.16 × 10^−3^
neuron apoptotic process	GO:0051402	14	[BAX, FYN, HSPA5, KCNB1, LRP1, MSH2, PARP1, PCSK9, PSEN1, RB1, RHOA, ROCK1, SCN2A, TFRC]	4.50	2.47 × 10^−5^
positive regulation of neuron death	GO:1901216	7	[BAX, CALM2, FYN, PARP1, PCSK9, PICALM, RHOA]	5.98	5.27 × 10^−4^
Schwann cell differentiation	GO:0014037	3	[LAMB2, RELA, VTA1]	6.00	2.21 × 10^−2^
astrocyte development	GO:0014002	5	[EGFR, GFAP, LAMB2, LRP1, PSEN1]	8.33	7.58 × 10^−4^
regulation of ERBB signalling pathway	GO:1901184	10	[CDC42, EGFR, EPN1, HGS, PDCD6IP, PSEN1, RAB7A, RTN4, SOCS5, SOS1]	7.87	3.09 × 10^−6^
negative regulation of neuron projection development	GO:0010977	9	[ARF6, GFAP, LRP1, MAPK1, PPP3CA, PSEN1, RHOA, RTN4, THY1]	4.59	6.15 × 10^−4^
positive regulation of neuron projection development	GO:0010976	18	[AP2A1, BMX, DDX21, FN1, FYN, HSPA5, HSPA8, ITGA3, KDM1A, LRP1, NME2, PDCD6IP, PSEN1, RAB21, RHOA, SCARB2, SOS1, TMEM30A]	4.75	7.81 × 10^−7^
regulation of p38MAPK cascade	GO:1900744	3	[DLG1, PDCD6IP, SOS1]	6.00	2.21 × 10^−2^
regulation of glutamate receptor signalling pathway	GO:1900449	5	[DLG1, FYN, HSPA8, MAPK1, NETO2]	5.49	4.79 × 10^−3^
non-canonical Wnt signalling pathway	GO:0035567	16	[AP2A1, AP2A2, AP2B1, CALM2, CAMK2A, CDC42, PDCD6IP, PICALM, PLCB3, PPP3CA, PSMA1, PSMA3, PSMB9, PSMD4, RAC1, RHOA]	8.56	9.57 × 10^−10^
negative regulation of cell morphogenesis involved in differentiation	GO:0010771	7	[MAPK1, POSTN, PPP3CA, PSEN1, RHOA, RTN4, THY1]	5.69	7.11 × 10^−4^
positive regulation of cell morphogenesis involved in differentiation	GO:0010770	8	[ARPC2, CDC42, FN1, LRP1, NCOA3, RAB21, RAC1, RHOA]	4.12	2.41 × 10^−3^
negative regulation of axonogenesis	GO:0050771	5	[MAPK1, PSEN1, RHOA, RTN4, THY1]	5.32	5.49 × 10^−3^
positive regulation of plasma membrane bounded cell projection assembly	GO:0120034	7	[ARPC2, CDC42, FN1, FSCN1, LAP3, PICALM, RAC1]	4.64	2.33 × 10^−3^
neuron projection fasciculation	GO:0106030	3	[BMX, MAPK1, RTN4]	8.57	8.40 × 10^−3^
neuron projection organization	GO:0106027	6	[CDC42, CTTN, FYN, ITGA3, MAPK1, PSEN1]	4.62	4.84 × 10^−3^
neural nucleus development	GO:0048857	7	[ACTB, CALM2, CDC42, HSPA5, PICALM, POTEF, RHOA]	7.69	1.12 × 10^−4^
regulation of neurotransmitter receptor activity	GO:0099601	5	[ATXN2, DLG1, HSPA8, MAPK1, NETO2]	4.72	9.03 × 10^−3^
dendrite morphogenesis	GO:0048813	9	[CALM2, CAMK2A, CDC42, FYN, HPRT1, MAPK1, PICALM, PPP3CA, RAB21]	4.48	7.36 × 10^−4^
postsynapse organization	GO:0099173	12	[ACTB, ARF4, ARF6, CDC42, CTTN, DLG1, FYN, ITGA3, MAPK1, PDCD6IP, POTEF, VTA1]	5.22	2.26 × 10^−5^
astrocyte differentiation	GO:0048708	8	[EGFR, GFAP, KRT7, LAMB2, LRP1, MAPK1, PSEN1, PTPN11]	7.27	5.41 × 10^−5^
regulation of postsynaptic membrane neurotransmitter receptor levels	GO:0099072	5	[AP2B1, CTNND1, DLG1, HSP90AA1, HSPA8]	5.56	4.57 × 10^−3^
response to axon injury	GO:0048678	5	[ARF4, BAX, GNAI2, LAMB2, LRP1]	5.15	9.03 × 10^−3^
axon extension	GO:0048675	7	[CTTN, FN1, HSP90AA1, LAMB2, LRP1, RAB21, RTN4]	4.70	2.70 × 10^−2^
regulation of action potential	GO:0098900	5	[CACNB3, CAMK2D, CAV1, DLG1, KCNB1]	7.04	3.59 × 10^−4^
thymus development	GO:0048538	3	[MAPK1, PRKDC, PSEN1]	4.92	3.57 × 10^−5^
synaptic vesicle endocytosis	GO:0048488	6	[ACTB, ARF6, CALM2, PICALM, POTEF, ROCK1]	8.33	1.08 × 10^−9^
glial cell differentiation	GO:0010001	13	[EGFR, GFAP, GSTP1, KRT7, LAMB2, LRP1, MAPK1, PSEN1, PSMD4, PTPN11, RELA, RHOA, VTA1]	4.74	2.32 × 10^−2^
regulation of fibroblast proliferation	GO:0048145	10	[ANXA2, BAX, EGFR, FN1, GSTP1, MIF, PDCD6IP, PML, PPP3CA, PRKDC]	8.06	2.09 × 10^−2^
receptor signalling pathway via STAT	GO:0097696	10	[CAV1, FGFR3, FYN, HGS, IL3, JAK3, KLK3, SOCS5, STAT1, TFRC]	4.67	1.90 × 10^−8^
astrocyte activation	GO:0048143	3	[EGFR, LRP1, PSEN1]	9.38	5.94 × 10^−2^
calcium ion transmembrane import into cytosol	GO:0097553	9	[ATP2B4, BAX, CALM2, CAMK2D, FYN, PICALM, PLCB3, TFRC, THY1]	4.46	2.60 × 10^−3^
neurotrophin TRK receptor signalling pathway	GO:0048011	3	[CTNND1, PTPN11, SOS1]	6.38	1.73 × 10^−3^
dendritic spine organization	GO:0097061	5	[CDC42, CTTN, FYN, ITGA3, MAPK1]	4.39	1.09 × 10^−3^
fibroblast growth factor receptor signalling pathway	GO:0008543	8	[CCN2, FGF21, FGF3, FGFR3, MAPK1, POSTN, PTPN11, THBS1]	6.15	1.99 × 10^−3^
glial cell migration	GO:0008347	5	[LRP1, PDCD6IP, RTN4, SUN1, SYNE2]	6.58	3.69 × 10^−2^
regulation of receptor signalling pathway via JAK-STAT	GO:0046425	8	[CAV1, FGFR3, FYN, HGS, IL3, JAK3, KLK3, TFRC]	4.37	2.26 × 10^−4^
neuron recognition	GO:0008038	3	[BMX, MAPK1, RTN4]	4.55	5.98 × 10^−4^
neuron migration	GO:0001764	9	[BAX, CAMK2A, ELP3, FYN, ITGA3, NAV1, PDCD6IP, PSEN1, RAC1]	4.39	1.75 × 10^−4^
endoderm development	GO:0007492	5	[BPTF, FN1, ITGAV, LAMB2, TFAP2C]	5.62	5.19 × 10^−4^
axonal fasciculation	GO:0007413	3	[BMX, MAPK1, RTN4]	8.57	1.39 × 10^−2^
axonogenesis	GO:0007409	24	[BMX, CALM2, CTTN, FAM129B, FLOT1, FN1, FYN, GRB7, HSP90AA1, LAMB2, LRP1, MAPK1, PICALM, PSEN1, PTPN11, RAB21, RAC1, RHOA, RTN4, SOS1, SPTBN1, THY1, TUBB3, UCHL1]	4.05	1.13 × 10^−2^
tyrosine phosphorylation of STAT protein	GO:0007260	5	[CAV1, FGFR3, FYN, IL3, JAK3]	4.72	1.11 × 10^−3^
receptor signalling pathway via JAK-STAT	GO:0007259	10	[CAV1, FGFR3, FYN, HGS, IL3, JAK3, KLK3, SOCS5, STAT1, TFRC]	4.81	2.74 × 10^−3^
Wnt signalling pathway, calcium modulating pathway	GO:0007223	5	[CALM2, CAMK2A, PICALM, PLCB3, PPP3CA]	9.80	1.94 × 10^−6^
glutamate receptor signalling pathway	GO:0007215	6	[DLG1, FYN, HSPA8, KCNB1, MAPK1, NETO2]	4.62	4.76 × 10^−4^
epidermal growth factor receptor signalling pathway	GO:0007173	15	[ARF4, CDC42, CTNND1, EFEMP1, EGFR, EPN1, GRB7, HGS, HSP90AA1, PDCD6IP, PSEN1, PTPN11, RAB7A, SOCS5, SOS1]	9.20	3.60 × 10^−7^

**Table 3:** Proteins involved in the proposed VPA induction pathway of neural differentiation of ADSCs.

Protein Name	Gene	Accession Numbers	Molecular Weight (Da)	Identification Probability	Gene Ontology
Signal transducer and activator of transcription 1-alpha/beta	STAT1	P42224	87,336.90	100%	GO:0007259
Calcium-binding mitochondrial carrier protein SCaMC-1	SLC25A24	Q6NUK1-2	53,356.60	92.60%	GO:0034599
Fibroblast growth factor 21	FGF21	Q9NSA1	22,300.60	92.50%	GO:0090080
Advillin	AVIL	O75366-2	92,029.10	92.20%	GO:0007399
Potassium channel subfamily T member 2	KCNT2	Q6UVM3-2, Q6UVM3-3	130,506.30	89.50%	GO:0005249
Mitogen-activated protein kinase 1	MAPK1	P28482-2	41,391.90	86.90%	GO:0000165
Neuropilin and tolloid-like protein 2	NETO2	Q8NC67-3	59,393.90	85.80%	GO:2000312
Tyrosine-protein kinase JAK3	JAK3	P52333-2	125,101.70	81.90%	GO:0046425
Sodium/calcium exchanger 3	SLC8A3	P57103-2, P57103-6, P57103-7	103,011.70	77.90%	GO:0060291
Signal transducer and activator of transcription 6	STAT6	P42226	94,136.90	59.90%	GO:0019221
Glial fibrillary acidic protein	GFAP	P14136-2, P14136-3	49,881.40	35.40%	GO:0031102
Potassium voltage-gated channel subfamily B member 1	KCNB1	Q14721	95,881.40	14.40%	GO:1900454
Potassium voltage-gated channel subfamily G member 4	KCNG4	Q8TDN1	58,981.00	11.80%	GO:0005251
Fibroblast growth factor receptor 3	FGFR3	P22607-2	877,100.00	6.20%	GO:0043410

## Abbreviations

ADSCs	Adipose Derived Stem Cells
ALP	Alkaline phosphatase
AVIL	Advillin
BHA	butylated hydroxyanisole
BME	beta-mercaptoethanol
DMEM	Dulbecco’s Modified Eagle’s Medium: Nutrient Mixture F-12
DMSO	dimethylsulfoxide
ERK	Extracellular Receptor Kinase
FGF21	Fibroblast growth factor 21
FGFR3	Fibroblast growth factor receptor 3
GFAP	Glial fibrillary acidic protein
hADSCs	human Adipose Derived Stem Cells
HDAC	histone deacetylase
JAK	Janus kinase
JAK3	Tyrosine-protein kinase JAK3
KCNB1	Potassium voltage-gated channel subfamily B member 1
KCNG4	Potassium voltage-gated channel subfamily G member 4
KCNT2	Potassium channel subfamily T member 2
LC-MS/MS	Liquid chromatography tandem mass spectrometry
MAPK1	Mitogen-activated protein kinase
NETO2	Neuropilin and tolloid-like protein 2
PBS	Phosphate-buffered saline
RA	Retinoic acid
SLC25A24	Calcium-binding mitochondrial carrier protein SCaMC-1
SLC8A3	Sodium/calcium exchanger 3
STAT1	Signal transducer and activator of transcription 1-alpha/beta
STAT6	Signal transducer and activator of transcription 6
VPA	Valproic Acid

## References

[1] Franco Lambert A.P., Fraga Zandonai A., Bonatto D., Cantarelli Machado D., Pegas Henriques J.A. (2009). Differentiation of human adipose-derived adult stem cells into neuronal tissue: Does it work?. Differ. Res. Biol. Divers..

[2] Woodbury D., Schwarz E.J., Prockop D.J., Black I.B. (2000). Adult rat and human bone marrow stromal cells differentiate into neurons. J. Neurosci. Res..

[3] Santos J., Milthorpe B.K., Herbert B.R., Padula M.P. (2017). Proteomic Analysis of Human Adipose Derived Stem Cells during Small Molecule Chemical Stimulated Pre-neuronal Differentiation. Int. J. Stem Cells.

[4] Kondo T., Johnson S.A., Yoder M.C., Romand R., Hashino E. (2005). Sonic hedgehog and retinoic acid synergistically promote sensory fate specification from bone marrow-derived pluripotent stem cells. Proc. Natl. Acad. Sci. USA.

[5] Yu J.M., Bunnell B.A., Kang S.K. (2011). Neural differentiation of human adipose tissue-derived stem cells. Methods Mol. Biol. (Clifton, N.J.).

[6] Mu M.W., Zhao Z.Y., Li C.G. (2015). Comparative study of neural differentiation of bone marrow mesenchymal stem cells by different induction methods. Genet. Mol. Res. GMR.

[7] Santos J., Milthorpe B.K., Padula M.P. (2019). Proteomic Analysis of Cyclic Ketamine Compounds Ability to Induce Neural Differentiation in Human Adult Mesenchymal Stem Cells. Int. J. Mol. Sci..

[8] Talwadekar M., Fernandes S., Kale V., Limaye L. (2017). Valproic acid enhances the neural differentiation of human placenta derived-mesenchymal stem cells in vitro. J. Tissue Eng. Regen. Med..

[9] Vukicevic V., Qin N., Balyura M., Eisenhofer G., Wong M.L., Licinio J., Bornstein S.R., Ehrhart-Bornstein M. (2015). Valproic acid enhances neuronal differentiation of sympathoadrenal progenitor cells. Mol. Psychiatry.

[10] Eckschlager T., Plch J., Stiborova M., Hrabeta J. (2017). Histone Deacetylase Inhibitors as Anticancer Drugs. Int. J. Mol. Sci..

[11] Gottlicher M., Minucci S., Zhu P., Kramer O.H., Schimpf A., Giavara S., Sleeman J.P., Lo Coco F., Nervi C., Pelicci P.G. (2001). Valproic acid defines a novel class of HDAC inhibitors inducing differentiation of transformed cells. EMBO J..

[12] Yu I.T., Park J.-Y., Kim S.H., Lee J.-S., Kim Y.-S., Son H. (2009). Valproic acid promotes neuronal differentiation by induction of proneural factors in association with H4 acetylation. Neuropharmacology.

[13] Hsieh J., Nakashima K., Kuwabara T., Mejia E., Gage F.H. (2004). Histone deacetylase inhibition-mediated neuronal differentiation of multipotent adult neural progenitor cells. Proc. Natl. Acad. Sci. USA.

[14] Liu X.S., Chopp M., Kassis H., Jia L.F., Hozeska-Solgot A., Zhang R.L., Chen C., Cui Y.S., Zhang Z.G. (2012). Valproic acid increases white matter repair and neurogenesis after stroke. Neuroscience.

[15] Rezaei F., Tiraihi T., Abdanipour A., Hassoun H.K., Taheri T. (2018). Immunocytochemical analysis of valproic acid induced histone H3 and H4 acetylation during differentiation of rat adipose derived stem cells into neuron-like cells. Biotech. Histochem..

[16] Long X., Olszewski M., Huang W., Kletzel M. (2005). Neural cell differentiation in vitro from adult human bone marrow mesenchymal stem cells. Stem Cells Dev..

[17] Tomita M., Mori T., Maruyama K., Zahir T., Ward M., Umezawa A., Young M.J. (2006). A comparison of neural differentiation and retinal transplantation with bone marrow-derived cells and retinal progenitor cells. Stem Cells (Dayton, Ohio).

[18] Galindo L.T., Filippo T.R.M., Semedo P., Ariza C.B., Moreira C.M., Camara N.O.S., Porcionatto M.A. (2011). Mesenchymal Stem Cell Therapy Modulates the Inflammatory Response in Experimental Traumatic Brain Injury. Neurol. Res. Int..

[19] Štefková K., Procházková J., Pacherník J. (2015). Alkaline phosphatase in stem cells. Stem Int..

[20] Hanna H., Mir L.M., Andre F.M. (2018). In vitro osteoblastic differentiation of mesenchymal stem cells generates cell layers with distinct properties. Stem Cell Res. Ther..

[21] Taverner T., Karpievitch Y.V., Polpitiya A.D., Brown J.N., Dabney A.R., Anderson G.A., Smith R.D. (2012). DanteR: An extensible R-based tool for quantitative analysis of -omics data. Bioinformatics.

[22] Shannon P., Markiel A., Ozier O., Baliga N.S., Wang J.T., Ramage D., Amin N., Schwikowski B., Ideker T. (2003). Cytoscape: A software environment for integrated models of biomolecular interaction networks. Genome Res..

[23] Lu P., Blesch A., Tuszynski M.H. (2004). Induction of bone marrow stromal cells to neurons: Differentiation, transdifferentiation, or artifact?. J. Neurosci. Res..

[24] Wang W., Tan J., Xing Y., Kan N., Ling J., Dong G., Liu G., Chen H. (2016). p43 induces IP-10 expression through the JAK-STAT signaling pathway in HMEC-1 cells. Int. J. Mol. Med..

[25] Moshapa F.T., Riches-Suman K., Palmer T.M. (2019). Therapeutic Targeting of the Proinflammatory IL-6-JAK/STAT Signalling Pathways Responsible for Vascular Restenosis in Type 2 Diabetes Mellitus. Cardiol. Res. Pract..

[26] Jatiani S.S., Baker S.J., Silverman L.R., Reddy E.P. (2010). JAK/STAT Pathways in Cytokine Signaling and Myeloproliferative Disorders: Approaches for Targeted Therapies. Genes Cancer.

[27] Rezaie P., Trillo-Pazos G., Everall I.P., Male D.K. (2002). Expression of beta-chemokines and chemokine receptors in human fetal astrocyte and microglial co-cultures: Potential role of chemokines in the developing CNS. Glia.

[28] Choi S.S., Lee H.J., Lim I., Satoh J.-I., Kim S.U. (2014). Human Astrocytes: Secretome Profiles of Cytokines and Chemokines. PLoS ONE.

[29] Wang T., Yuan W., Liu Y., Zhang Y., Wang Z., Zhou X., Ning G., Zhang L., Yao L., Feng S. (2015). The role of the JAK-STAT pathway in neural stem cells, neural progenitor cells and reactive astrocytes after spinal cord injury. Biomed. Rep..

[30] Boehme M., Guenther M., Stahr A., Liebmann M., Jaenisch N., Witte O.W., Frahm C. (2014). Impact of indomethacin on neuroinflammation and hippocampal neurogenesis in aged mice. Neurosci. Lett..

[31] Koo J.W., Duman R.S. (2008). IL-1β is an essential mediator of the antineurogenic and anhedonic effects of stress. Proc. Natl. Acad. Sci. USA.

[32] Monje M.L., Toda H., Palmer T.D. (2003). Inflammatory blockade restores adult hippocampal neurogenesis. Science.

[33] Johansson S., Price J., Modo M. (2008). Effect of Inflammatory Cytokines on Major Histocompatibility Complex Expression and Differentiation of Human Neural Stem/Progenitor Cells. Stem Cells (Dayton, Ohio).

[34] Chen E., Xu D., Lan X., Jia B., Sun L., Zheng J.C., Peng H. (2013). A Novel Role of the STAT3 Pathway in Brain Inflammation-induced Human Neural Progenitor Cell Differentiation. Curr. Mol. Med..

[35] Jain N., Zhang T., Fong S.L., Lim C.P., Cao X. (1998). Repression of Stat3 activity by activation of mitogen-activated protein kinase (MAPK). Oncogene.

[36] Kim M.-H., Kim M.-S., Kim W., Kang M.A., Cacalano N.A., Kang S.-B., Shin Y.-J., Jeong J.-H. (2015). Suppressor of Cytokine Signaling (SOCS) Genes Are Silenced by DNA Hypermethylation and Histone Deacetylation and Regulate Response to Radiotherapy in Cervical Cancer Cells. PLoS ONE.

[37] Cooney R.N. (2002). Suppressors of cytokine signaling (SOCS): Inhibitors of the JAK/STAT pathway. Shock (Augusta, Ga.).

[38] Sharma N., Nickl C., Kang H., Winter S.S., Cannon J., Matlawska-Wasowska K. (2017). SOCS5 Regulates JAK-STAT Signaling and T-ALL Development. Blood.

[39] Henson E.S., Gibson S.B. (2006). Surviving cell death through epidermal growth factor (EGF) signal transduction pathways: Implications for cancer therapy. Cell. Signal..

[40] Bonni A., Sun Y., Nadal-Vicens M., Bhatt A., Frank D.A., Rozovsky I., Stahl N., Yancopoulos G.D., Greenberg M.E. (1997). Regulation of gliogenesis in the central nervous system by the JAK-STAT signaling pathway. Science.

[41] Kario E., Marmor M.D., Adamsky K., Citri A., Amit I., Amariglio N., Rechavi G., Yarden Y. (2005). Suppressors of cytokine signaling 4 and 5 regulate epidermal growth factor receptor signaling. J. Biol. Chem..

[42] Tung E.W., Winn L.M. (2011). Valproic acid increases formation of reactive oxygen species and induces apoptosis in postimplantation embryos: A role for oxidative stress in valproic acid-induced neural tube defects. Mol. Pharmacol..

[43] Yuan T.-F., Gu S., Shan C., Marchado S., Arias-Carrión O. (2015). Oxidative Stress and Adult Neurogenesis. Stem Cell Rev. Rep..

[44] Okubo T., Fujimoto S., Hayashi D., Suzuki T., Sakaue M., Miyazaki Y., Tanaka K., Usami M., Takizawa T. (2019). Valproic acid promotes mature neuronal differentiation of adipose tissue-derived stem cells through iNOS–NO–sGC signaling pathway. Nitric Oxide.

[45] Han E.-S., Muller F.L., Pérez V.I., Qi W., Liang H., Xi L., Fu C., Doyle E., Hickey M., Cornell J. (2008). The in vivo gene expression signature of oxidative stress. Physiol. Genom..

[46] Traba J., Del Arco A., Duchen M.R., Szabadkai G., Satrustegui J. (2012). SCaMC-1 promotes cancer cell survival by desensitizing mitochondrial permeability transition via ATP/ADP-mediated matrix Ca(2+) buffering. Cell Death Differ..

[47] Mi H., Muruganujan A., Ebert D., Huang X., Thomas P.D. (2018). PANTHER version 14: More genomes, a new PANTHER GO-slim and improvements in enrichment analysis tools. Nucleic Acids Res..

[48] Kurata S.-I. (2000). Selective Activation of p38 MAPK Cascade and Mitotic Arrest Caused by Low Level Oxidative Stress. J. Biol. Chem..

[49] Leng Y., Wang J., Wang Z., Liao H.M., Wei M., Leeds P., Chuang D.M. (2016). Valproic Acid and Other HDAC Inhibitors Upregulate FGF21 Gene Expression and Promote Process Elongation in Glia by Inhibiting HDAC2 and 3. Int. J. Neuropsychopharmacol..

[50] Kuroda M., Muramatsu R., Maedera N., Koyama Y., Hamaguchi M., Fujimura H., Yoshida M., Konishi M., Itoh N., Mochizuki H. (2017). Peripherally derived FGF21 promotes remyelination in the central nervous system. J. Clin. Investig..

[51] Iwata T., Hevner R.F. (2009). Fibroblast growth factor signaling in development of the cerebral cortex. Dev. Growth Differ..

[52] Shahror R.A., Linares G.R., Wang Y., Hsueh S.C., Wu C.C., Chuang D.M., Chiang Y.H., Chen K.Y. (2019). Transplantation of Mesenchymal Stem Cells Overexpressing Fibroblast Growth Factor 21 Facilitates Cognitive Recovery and Enhances Neurogenesis in a Mouse Model of Traumatic Brain Injury. J. Neurotrauma.

[53] Miller F.D., Gauthier A.S. (2007). Timing Is Everything: Making Neurons versus Glia in the Developing Cortex. Neuron.

[54] Wei Z., Sun M., Liu X., Zhang J., Jin Y. (2014). Rufy3, a protein specifically expressed in neurons, interacts with actin-bundling protein Fascin to control the growth of axons. J. Neurochem..

[55] Vernon C.G., Swanson G.T. (2017). Neto2 Assembles with Kainate Receptors in DRG Neurons during Development and Modulates Neurite Outgrowth in Adult Sensory Neurons. J. Neurosci. Off. J. Soc. Neurosci..

